# Mitochondrion-mediated cell death: dissecting yeast apoptosis for a better understanding of neurodegeneration

**DOI:** 10.3389/fonc.2012.00182

**Published:** 2012-11-28

**Authors:** Ralf J. Braun

**Affiliations:** Institut für Zellbiologie, Universität BayreuthBayreuth, Germany

**Keywords:** mitochondria, mitochondrial dysfunction, mitochondrial quality control, neurodegeneration, neurotoxicity, cell death, *Saccharomyces cerevisiae*

## Abstract

Mitochondrial damage and dysfunction are common hallmarks for neurodegenerative disorders, including Alzheimer, Parkinson, Huntington diseases, and the motor neuron disorder amyotrophic lateral sclerosis. Damaged mitochondria pivotally contribute to neurotoxicity and neuronal cell death in these disorders, *e.g*., due to their inability to provide the high energy requirements for neurons, their generation of reactive oxygen species (ROS), and their induction of mitochondrion-mediated cell death pathways. Therefore, in-depth analyses of the underlying molecular pathways, including cellular mechanisms controlling the maintenance of mitochondrial function, is a prerequisite for a better understanding of neurodegenerative disorders. The yeast *Saccharomyces cerevisiae* is an established model for deciphering mitochondrial quality control mechanisms and the distinct mitochondrial roles during apoptosis and programmed cell death. Cell death upon expression of various human neurotoxic proteins has been characterized in yeast, revealing neurotoxic protein-specific differences. This review summarizes how mitochondria are affected in these neurotoxic yeast models, and how they are involved in the execution and prevention of cell death. I will discuss to which extent this mimics the situation in other neurotoxic model systems, and how this may contribute to a better understanding of the mitochondrial roles in the human disorders.

## Introduction

Mitochondria are important organelles; they produce most of the ATP via oxidative phosphorylation, they are involved in lipid and phospholipid metabolism, in the biosynthesis of essential intermediates, including heme and iron-sulfur clusters, and they contribute to various cellular stress responses, including programmed cell death (Nunnari and Suomalainen, 2012). Functional mitochondria are essential for neurons, which have an extreme high demand for energy in their cell bodies and synapses (Nunnari and Suomalainen, 2012). Therefore, it is evident that neurons are extremely vulnerable against mitochondrial damage.

**Figure 1:**
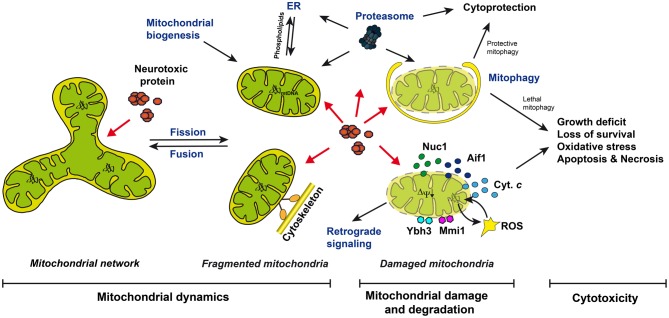
**Mitochondrial dynamics, damage and degradation, and mitochondrion-dependent cell death upon neurotoxic protein expression in yeast.** Mitochondria are part of a network promoted by fusion or are fragmented into individual organelles by fission. They are transported along the cytoskeleton. Damaged mitochondria are targeted by cytoplasmic proteins, including Ybh3 and Mmi1, produce ROS or release proteins into the cytosol, including cytochrome *c*, Aif1, and Nuc1, triggering apoptosis and necrosis. Mitophagy and proteasome-dependent pathways remove damaged mitochondria, and mitochondrial biogenesis and retrograde signaling are involved in the replenishment and repair of the mitochondrial pool, respectively. Neurotoxic proteins trigger mitochondrial damage and cell death: they could interfere with mitochondrial fusion, fission, and motility, or could interrupt with mitochondrion degradation triggering “lethal mitophagy”, or could directly affect mitochondrial function.

Mitochondrial damage is a hallmark for Alzheimer, Parkinson, Huntington diseases, and amyotrophic lateral sclerosis (Martin, 2011; Correia et al., 2012; Cozzolino et al., 2012). Affected neurons suffer from ATP depletion, loss of respiratory capacity, elevated levels of reactive oxygen species (ROS), the induction of mitochondrion-specific cell death pathways, and immotile mitochondria, which fail to localize to the sites with increased energy demands, such as synapses (Martin, 2011; Correia et al., 2012; Cozzolino et al., 2012) (Figure 1). Mitochondrial damage in neurons can be described by (1) mutations in the mitochondrial DNA (mtDNA), (2) the loss of the mitochondrial membrane potential, (3) the loss of mitochondrial protein import and protein biosynthesis, (4) reduced activities of enzymes of the mitochondrial respiratory chain and the TCA cycle, (5) increased leakage of electrons from the respiratory chain generating ROS, (6) the loss of mitochondrial motility, (7) the destruction of the mitochondrial network, and (8) the rupture of the mitochondrial outer and inner membranes, leading (9) to the release of mitochondrial pro-death factors, including cytochrome *c* (Cyt. *c*), apoptosis-inducing factor, or endonuclease G.

Nature developed many mechanisms to prevent and repair mitochondrial damage or to remove damaged mitochondria (Figure 1): (1) the dynamic fusion and fission of mitochondria enables the maintenance of intact mtDNA (Westermann, 2010; Hori et al., 2011), (2) the phospholipid turnover between the ER and mitochondria allows for the rejuvenation of mitochondrial membranes (Fujimoto and Hayashi, 2011), (3) retrograde signaling senses mitochondrial damage, eliciting a nuclear transcriptional response to counteract mitochondrial damage (Liu and Butow, 2006; Pellegrino et al., 2012), (4) mitochondrial biogenesis leads to the replenishment of damaged mitochondria and its components (Michel et al., 2012), (5) mitochondrial protein quality control is guaranteed by chaperones and the proteolytic machinery, including the proteasome-dependent mitochondrion-associated degradation (MAD) of mitochondrial outer membrane proteins (Heo et al., 2010; Taylor and Rutter, 2011), the proteolytic degradation of proteins of the intermembrane space, the inner membrane, and the matrix (Fischer et al., 2012; Rugarli and Langer, 2012), and (6) removal of damaged mitochondria occurs via mitochondrion-specific autophagy (mitophagy) (Kanki and Klionsky, 2010). Thus, the detailed analysis of mitochondrial damage, and the mechanisms counteracting mitochondrial damage and its devastating consequences for neurons, is of high importance for a better understanding of neurodegenerative disorders.

Yeast is an established model for dissecting conserved mechanisms of programmed cell death including apoptosis and necrosis (Carmona-Gutierrez et al., 2010). In yeast, cytotoxicity can easily be measured by complementary methods (Braun et al., 2010). Growth assays measure growth rates on agar plates or in liquid cultures, clonogenic survival assays measure the ability of yeast cells to form new colonies, and necrosis and apoptosis can be discriminated by analyzing cells for morphological markers of these subroutines of cell death. The importance of mitochondria in distinct cell death scenarios is well described in yeast (Eisenberg et al., 2007; Pereira et al., 2008; Guaragnella et al., 2012) (Figure 1). The mitochondrial permeabilization and the consequent release of the mitochondrial cell death proteins Cyt. *c*, yeast apoptosis-inducing factor (Aif1), or yeast endonuclease G (Nuc1) was observed (Ludovico et al., 2002; Wissing et al., 2004; Büttner et al., 2007; Pereira et al., 2007). ROS are produced from the mitochondrial respiratory chain, including the internal NADH dehydrogenase (Ndi1) and complex III (Braun et al., 2006, 2011; Li et al., 2006). Cytoplasmic proteins are recruited to the mitochondrial outer membrane, such as the BH3-only protein Ybh3 and the microtubule-associated protein Mmi1 (Rinnerthaler et al., 2006; Büttner et al., 2011). Mitochondrial quality control mechanisms and their influence on cell survival and aging have been intensively examined in yeast (Westermann, 2010; Braun and Westermann, 2011; Müller and Reichert, 2011; Fischer et al., 2012; Nunnari and Suomalainen, 2012; Rugarli and Langer, 2012). Indeed, our knowledge about mitochondrial dynamics, including mitochondrial fusion, fission, motility, degradation, and protein quality control is strongly influenced by data originally obtained in yeast, and later validated in other organisms, including humans. In the recent years, many yeast models have been established to analyze the influence of human neurotoxic protein expression on yeast cell survival, including models for Alzheimer, Parkinson, Huntington, and motor neuron disorders (Gitler, 2008; Miller-Fleming et al., 2008; Winderickx et al., 2008; Bharadwaj et al., 2010; Braun et al., 2010; Khurana and Lindquist, 2010; Bastow et al., 2011; De Vos et al., 2011; Mason and Giorgini, 2011).

Here, I summarize how mitochondria are damaged in these neurotoxic yeast cell death models, which role they play in the execution of cell death, and which mitochondrial quality control mechanisms potentially influence cytotoxicity. Further, I will critically discuss the similarities and discrepancies between the neurotoxic yeast models, and the animal and cell culture disease models.

## Neurotoxic yeast models

### Alzheimer disease (AD) and frontotemporal lobar degeneration (FTLD-tau)

AD is the most prevalent form of age-related dementia (Querfurth and Laferla, 2010). It is characterized by a progressive deterioration, concomitant with behavior impairments and deficits in language and visuospatial skills (Querfurth and Laferla, 2010). Synaptic loss and neuronal decline can be observed in affected brain regions, including the hippocampus and the cortex (Querfurth and Laferla, 2010). The accumulation of extracellular senile plaques and intracellular aggregates, composed of the β-amyloid peptide, and the intracellular accumulation of neurofibrillary tangles comprising hyperphosphorylated variants of the microtubule-associated protein tau contribute to the progression of AD (Laferla et al., 2007; Benilova et al., 2012; Mandelkow and Mandelkow, 2012).

FTLD is the second most common form of early onset dementia after AD (Sieben et al., 2012). It is characterized by a selective atrophy of the frontal and anterior temporal lobes of the brain, leading to disturbances of behavior and personality (Sieben et al., 2012). Intracellular accumulation of the hyperphosphorylated protein tau is a hallmark of one subtype of FTLD, called FTLD-tau (Sieben et al., 2012). Mutations in *MAPT*, the gene encoding tau, trigger neuronal degeneration and FTLD-tau (Sieben et al., 2012). In order to elucidate conserved mechanisms of AD/FTLD-relevant cytotoxicity, yeast models expressing AD-associated β-amyloid, and AD/FTLD-associated wild-type and mutant tau were established (Bharadwaj et al., 2010; De Vos et al., 2011).

#### AD-associated β-amyloid triggers cytotoxicity and mitochondrial damage upon localization to the secretory pathway

Several yeast AD models for dissecting the cytotoxic role of intracellular β-amyloid have been established (Bharadwaj et al., 2010; Treusch et al., 2011; D'Angelo et al., 2012). Whereas cytosolic β-amyloid and β-amyloid fusion proteins did not exert a marked cytotoxicity on yeast, two recent studies demonstrated that directing β-amyloid to the secretory pathway resulted in significant cytotoxicity, as measured by growth assays (Treusch et al., 2011; D'Angelo et al., 2012) (Table 1). In these models β-amyloid oligomerization could be observed, and aggregation and growth deficits were increased expressing AD-associated mutant β-amyloid (Treusch et al., 2011; D'Angelo et al., 2012). Although β-amyloid-triggered cytotoxicity was elicited in the secretory pathway, there are some hints that mitochondria were damaged and involved in the execution of cytotoxicity (D'Angelo et al., 2012). β-amyloid-expressing cells demonstrated decreased growth rates on obligatory respiratory growth media, and the oxygen consumption was markedly reduced in these cells under these conditions. Since the decrease in oxygen consumption preceded the observed growth deficits, these data suggest that mitochondrial impairment is causative for β-amyloid-triggered cytotoxicity. In a genome-wide overexpression screen to identify enhancers and suppressors of β-amyloid-triggered cytotoxicity (growth assays), *PET111* and *SLS1* encoding two mitochondrion-associated translation regulators were found as enhancers, and *RTG3*, encoding a transcriptional activator of the retrograde response, a response to counteract mitochondrial damage (Liu and Butow, 2006), was identified as suppressor (Treusch et al., 2011). Further studies will have to show, in which way mitochondrial protein translation and the retrograde response are involved in modulating β-amyloid-triggered cytotoxicity. It will also be of interest to investigate how β-amyloid expression can result in the potential mitochondrial damage in yeast, and whether it induces apoptotic and/or necrotic cell death. Although part of β-amyloid, which was directed to the secretory pathway, was later found to be cytosolic (D'Angelo et al., 2012), it remains an open question whether β-amyloid directly or indirectly affects mitochondrial function, and therewith influence cell survival.

**Table 1:** **Cytotoxicity, mitochondrial damage and mitochondrial quality control in yeast models for neurodegeneration**.

**Yeast model**	**Disease protein**	**Cytotoxicity**	**Mitochondrial damage**	**Mitochondrial quality control**	**Remarks**	**References**
AD	β-Amyloid	Yes	Respiratory impairment	Beneficial retrograde response?	–	Treusch et al., 2011; D'Angelo et al., 2012
AD/FTLD-tau	Tau	No	n.d.	n.d.	Mitochondrial dysfunction and mitochondrially produced ROS contribute to tau aggregation	Vanhelmont et al., 2010; De Vos et al., 2011
	Tau/α-synuclein	Yes	n.d.	n.d.	Synergistic cytotoxicity (growth impairment) upon co-expression of tau and α-synuclein	Zabrocki et al., 2005
PD	α-Synuclein	Yes	Mitochondrially produced ROS, mitochondrial fragmentation, mitochondrial swelling, cytochrome *c* release, loss of mitochondrial membrane potential	Lethal mitophagy, beneficial retrograde response? beneficial mitochondrial biogenesis?	No direct interaction between α-synuclein and mitochondria; impaired ER homeostasis may contribute to mitochondrial damage	Willingham et al., 2003; Flower et al., 2005; Büttner et al., 2008; Gitler et al., 2008; Lee et al., 2008; Yeger-Lotem et al., 2009; Su et al., 2010; Sampaio-Marques et al., 2012
	Lrrk2	Yes	n.d.	n.d.	Abnormal autophagic vacuoles	Xiong et al., 2010
	Ypk9 (ATP13A2)	No	n.d.	n.d.	Rescues α-synuclein- and manganese-triggered cytotoxicity; upon deletion mitochondrion-dependent hypersensitivity against manganese treatment	Gitler et al., 2009; Chesi et al., 2012
	Hsp31 (DJ-1)	n.d.	n.d.	n.d.	Upon deletion increased ROS levels, and hypersensitivity against oxidative stress	Skoneczna et al., 2007
HD	Huntingtin	Yes	Mitochondrially produced ROS, respiratory impairment, mitochondrial fragmentation and swelling, loss of mitochondrial membrane potential, decreased mitochondrial protein synthesis, accumulation of intermediates of the kynurenine pathway	Beneficial mitochondrial biogenesis, beneficial retrograde response?	Direct interaction of Huntingtin with mitochondria; impaired ER homeostasis may contribute to mitochondrial damage	Willingham et al., 2003; Giorgini et al., 2005, 2008; Sokolov et al., 2006; Solans et al., 2006; Wang et al., 2009; Ocampo et al., 2010; Mason and Giorgini, 2011; Tauber et al., 2011
ALS	SOD1	n.d.	Respiratory impairment	n.d.	Increased localization of ALS-associated SOD1 in the mitochondrial intermembrane space	Gunther et al., 2004; Klöppel et al., 2010; Bastow et al., 2011
	TDP-43	Yes	Mitochondrially produced ROS, respiratory capacity determines cytotoxicity	n.d.	Peri-mitochondrial TDP-43-containing aggregate-like foci	Johnson et al., 2008; Braun et al., 2011
	FUS/TLS	Yes	n.d.	n.d.	Deletion of genes encoding mitochondrion-localized proteins increase FUS/TLS-triggered cytotoxicity	Sun et al., 2011

n.d.: not determined.

#### Oxidative stress and mitochondrial dysfunction enhance aggregation of AD/FTLD-associated wild-type and mutant tau in yeast

Expression of FTLD-associated wild-type and mutant (P301L) tau did not trigger cytotoxicity in yeast, although it increased growth deficits upon co-expression with PD-associated α-synuclein (Zabrocki et al., 2005) (Table 1). Human tau formed sarkosyl-insoluble aggregates, which were highly phosphorylated by the yeast tau kinases Mds1 and Pho85 (Vandebroek et al., 2005, 2006; Vanhelmont et al., 2010). Treatment with ferrous sulfate, which increases ROS production, resulted in a significant increase in pathological tau aggregates in yeast cells expressing tau (Vanhelmont et al., 2010). This phenomenon was increased with FTLD-associated mutant (P301L) tau compared to wild-type tau, and it was independent of the phosphorylation status of tau, suggesting that ROS-increased tau aggregation acted mainly in parallel to tau phosphorylation (Vanhelmont et al., 2010). Increased pathological tau aggregates were also observed in yeast cells lacking the mitochondrial antioxidant enzyme superoxide dismutase 2 (*sod2*Δ), and in yeast cells lacking mtDNA (*rim1*Δ) (Vanhelmont et al., 2010). These data suggest that mitochondrially localized ROS and mitochondrial dysfunction contribute to tau pathology in yeast (Vanhelmont et al., 2010; De Vos et al., 2011).

#### Yeast AD models recapitulate key features observed in animal AD models and in AD patients

Mitochondrial damage and dysfunction are characteristics of both familiar and sporadic AD (Correia et al., 2012). Decreased abundances and activities of the pyruvate dehydrogenase complex, the TCA cycle enzymes, and mitochondrial respiratory chain complexes were observed in AD patients, animal and cell culture AD models (Correia et al., 2012). These data point to decreased mitochondrial activities and a consequent loss in energy supply (Correia et al., 2012). Yeast cells expressing β-amyloid demonstrated decreased oxygen consumption and decreased growth rates on obligatory respiratory growth media (D'Angelo et al., 2012), mimicking the energy hypometabolism observed in AD patients and other model systems. In AD patients and higher AD models, high levels of lipid and protein oxidation, as well as high incidences of mtDNA mutations hint to increased levels of oxidative stress in affected neurons (Correia et al., 2012). Oxidative stress has been suggested to facilitate pathological aggregation of β-amyloid and tau, which are then believed to further damage mitochondria in a vicious cycle (Swerdlow et al., 2010; Leuner et al., 2012). Although it remains undetermined whether β-amyloid or tau expression in yeast triggers ROS production, chemically induced oxidative stress in yeast cells expressing tau markedly increased its conversion into pathological tau aggregates (Vanhelmont et al., 2010). Thus, high levels of oxidative stress, *e.g*., due to damaged mitochondria, may contribute to increased levels of pathological tau, accelerating cell death (De Vos et al., 2011).

Since β-amyloid was found to directly interact with mitochondria in higher AD models, these data suggest that at least part of the mitochondrial damage might be elicited by a detrimental interaction between the disease peptide and the organelle (Correia et al., 2012). In fact, in yeast β-amyloid was also identified in the cytosol (D'Angelo et al., 2012). Whether a direct interaction between β-amyloid and mitochondria occurs remains unknown. In animal and cell culture AD/FTLD models expressing AD-associated β-amyloid or FTLD-associated tau, mitochondrial dynamics was demonstrated to be aberrantly altered: abnormal mitochondrial morphology, and altered mitochondrial fission and fusion, as well as decreased mitochondrial motility and increased mitophagy were observed (Correia et al., 2012; Schulz et al., 2012). Since the pathways regulating mitochondrial dynamics and mitochondrial quality control are conserved from yeast to humans (Westermann, 2010; Fischer et al., 2012; Rugarli and Langer, 2012), yeast may help to elucidate how these pathways influence cytotoxicity upon expression of β-amyloid or tau.

### Parkinson disease (PD)

PD is the most prevalent age-related movement disorder characterized by a progressive loss of dopaminergic neurons in the substantia nigra pars compacta (Lees et al., 2009). This results in a loss of the neurotransmitter dopamine in the striatum and consequently impairs with normal motor function leading to resting tremor, bradykinesia and rigidity (Lees et al., 2009). In most familiar and sporadic cases, PD is associated with Lewy bodies, *i.e*., intracellular cytoplasmic aggregates composed of the protein α-synuclein (Uversky, 2007). Consistently, missense mutations in the *SNCA* gene, resulting in the expression of α-synuclein variants (A30P, A53T, E46K), as well as duplication and triplication of *SNCA*, leading to elevated α-synuclein levels, are causative for PD in some familiar forms of the disorder (Uversky, 2007). Mutations in other genes, including genes encoding the leucine-rich repeat kinase 2 (LRRK2), the E3 ubiquitin ligase parkin, the chaperone DJ-1, the mitochondrial PTEN-induced putative kinase 1 (PINK1), and the lysosomal ATPase ATP13A2 also lead to PD (Dehay and Bezard, 2011). Numerous disease models either overexpressing or deleting these disease-associated genes have been established, in order to describe common and distinct pathways relevant for PD progression (Dehay and Bezard, 2011).

#### Overexpression of α-synuclein in yeast results in cytotoxicity, and mitochondrion-dependent programmed cell death

Yeast cells overexpressing wild-type and disease-associated α-synuclein demonstrated growth deficits and age-dependent loss of clonogenic cell survival (Willingham et al., 2003; Flower et al., 2005; Witt and Flower, 2006; Büttner et al., 2008; Lee et al., 2008) (Table 1). α-Synuclein-triggered cytotoxicity in yeast was characterized by increased levels of ROS, and by the emergence of morphological markers of both apoptosis and necrosis (Flower et al., 2005; Büttner et al., 2008; Su et al., 2010). Mitochondria are critically damaged upon α-synuclein expression: (1) The mitochondrial network was fragmented (Sampaio-Marques et al., 2012), (2) mitochondria were found to be abnormally swollen (Su et al., 2010), (3) Cyt. *c* was released from mitochondria into the cytosol (Flower et al., 2005), (4) α-synuclein-expressing cells treated with the proteasome inhibitor lactacystin demonstrated loss of mitochondrial membrane potential (Lee et al., 2008), and (5) mRNA profiling revealed that 60% of the downregulated genes encode proteins localized to mitochondria (Yeger-Lotem et al., 2009). Mitochondrial damage pivotally contributes to α-synuclein-triggered cytotoxicity, because α-synuclein expression in ρ^0^ cells, which lack mtDNA and are devoid of respiratory competent mitochondria, significantly relieved the loss of cell survival, reduced the number of apoptotic and necrotic cells and markedly decreased ROS levels (Büttner et al., 2008). Mitochondrially produced ROS are important in α-synuclein-triggered cytotoxicity: (1) Deletion of *SOD2* encoding the mitochondrial antioxidant enzyme superoxide dismutase 2 markedly increased α-synuclein-triggered growth deficits (Willingham et al., 2003), (2) α-synuclein-expressing yeast cells were hypersensitive against oxidative stress (clonogenic cell survival assay upon hydrogen peroxide treatment) (Flower et al., 2005), and (3) α-synuclein-triggered ROS accumulation could efficiently be suppressed with the antioxidant glutathione (Flower et al., 2005). In another study treatment of α-synuclein-expressing cells with the antioxidants *N*-acetylcysteine, riboflavin, and melatonin did not show marked suppression of cytotoxicity (plasma membrane integrity and growth assays) (Su et al., 2010), suggesting that ROS rather accelerate α-synuclein-triggered cytotoxicity but are not essential for it.

#### Indirect mechanisms result in mitochondrial damage upon α-synuclein expression in yeast

α-Synuclein did not localize to yeast mitochondria, neither in cultures with intermediate nor in cultures with high expression levels of this protein (Gitler et al., 2008; Su et al., 2010). These data suggest that α-synuclein does not directly induce mitochondrial damage by mislocalizing to this organelle, as was discussed earlier (Witt and Flower, 2006), but that mitochondria are impaired in more indirect ways (Su et al., 2010). In fact, high expression levels of α-synuclein triggered defects in ER homeostasis in yeast (Gitler et al., 2008). This could have detrimental effects on the phospholipid turnover from the ER to and from the mitochondrial membranes (Fujimoto and Hayashi, 2011), thereby damaging mitochondrial membranes (Su et al., 2010). Impaired ER trafficking could also impair mitophagy, resulting in the accumulation of damaged mitochondria (Su et al., 2010). Further studies will be needed to shed light into cellular mechanisms affecting yeast mitochondria during α-synuclein expression.

#### Mitophagy determines α-synuclein-triggered cytotoxicity in yeast

Using an approach combining data from mRNA profiling of α-synuclein-expressing cultures with data from genetic suppressor screens searching for modifiers of α-synuclein-triggered cytotoxicity (growth assays) predicted the target of rapamycin (TOR) pathway, as a modulator of α-synuclein-triggered cytotoxicity (Yeger-Lotem et al., 2009). Indeed, addition of the TOR-inhibitor rapamycin markedly enhanced the growth deficits elicited by α-synuclein (Yeger-Lotem et al., 2009). Since inactivation of the TOR pathway induces autophagy-related pathways, these data gave first hints, that enhancing autophagy could be harmful for cultures expressing α-synuclein. Consistently, both rates of autophagy and mitochondrion-specific autophagy (mitophagy) were significantly increased upon α-synuclein expression (Sampaio-Marques et al., 2012). Pharmacological inhibition of autophagy (and mitophagy) by treatment with chloroquine markedly extended chronological life span of yeast cells expressing α-synuclein (Sampaio-Marques et al., 2012). Notably, inhibition of mitophagy alone was sufficient to relieve α-synuclein-triggered cytotoxicity (Sampaio-Marques et al., 2012): yeast cultures lacking the mitophagy-specific genes *ATG11* and *ATG32* demonstrated loss of mitophagy upon expression of α-synuclein, concomitant to markedly increased chronological life spans, significantly reduced incidences of morphological and metabolic markers of cell death, markedly decreased ROS levels, and the restoration of the mitochondrial network. These data suggest that mitophagy, a *per se* protective pathway to remove damaged mitochondria, can exert lethal functions in yeast upon high levels of α-synuclein.

Inhibition of the mitochondrial retrograde response by overexpressing its negative regulator Mks1 enhanced α-synuclein-triggered growth deficits (Yeger-Lotem et al., 2009). In contrast, overexpression of *HAP4*, encoding a transcriptional activator of mitochondrial biogenesis genes, markedly suppressed α-synuclein-triggered growth deficits (Yeger-Lotem et al., 2009). Thus, the retrograde response and mitochondrial biogenesis potentially play protective roles in cultures upon high levels of α-synuclein, whereas mitophagy potentially exert a lethal function. Further studies are needed to discriminate the distinct roles of these different mitochondrial quality control mechanisms in modulating α-synuclein-triggered cytotoxicity.

#### Dissecting mitochondrial roles in other yeast PD models remains a future task

Yeast cultures expressing fragments of the PD-associated GTPase and protein kinase Lrrk2 demonstrated cytotoxicity as manifested as growth deficits (Xiong et al., 2010). Expression of the GTPase domain was sufficient to induce Lrrk2-triggered cytotoxicity (growth assays) (Xiong et al., 2010) (Table 1). PD-associated mutant variants with altered GTPase activity demonstrated markedly increased cytotoxicity when compared with cultures expressing wild-type GTPase fragments, underlining the notion that altered GTPase activity of Lrrk2 is an important factor determining neuronal cell loss during PD progression (Gloeckner et al., 2006; Xiong et al., 2010). Remarkably, expression of Lrrk2 GTPase variants resulted in a significant increase in autophagic vacuoles in yeast, and deletion of *GCN4*, encoding a transcriptional activator of autophagy genes, led to a marked decrease in Lrrk2-triggered cytotoxicity (growth assays) (Xiong et al., 2010). These data suggest that, like in yeast cells expressing α-synuclein, autophagy potentially plays detrimental roles in Lrrk2-induced cytotoxicity. However, whether mitophagy and mitochondrial damage are important in modulating Lrrk2-triggered cytotoxicity in yeast remain to be explored.

Expression of the vacuolar protein Ypk9 (yeast PARK9), the yeast homolog of the PD-associated lysosomal ATPase ATP13A2, suppressed α-synuclein-triggered cytotoxicity (growth assays) in yeast (Gitler et al., 2009) (Table 1). Ypk9 expression increased the cellular tolerance against elevated levels of manganese, which is an environmental risk factor for PD (Gitler et al., 2009). In contrast, deletion of *YPK9* resulted in cells demonstrating hypersensitivity against manganese treatment (growth assays) (Gitler et al., 2009; Chesi et al., 2012). This hypersensitivity was executed, at least in part, by functional mitochondria, as evidenced by the suppression of manganese-triggered cytotoxicity in yeast strains lacking genes encoding mitochondrial proteins, such as the GTPase Mgm1 and mitochondrial ribosomal proteins (Chesi et al., 2012). Further studies will have to show, whether Ypk9 expression prevents pivotal mitochondrial damage and aberrant mitophagy upon expression of α-synuclein.

The *S. cerevisiae* genome contains four homologs (*HSP31*, *HSP32*, *HSP33*, and *SNO4* (*HSP34*)) of the PD-associated gene DJ-1 (Skoneczna et al., 2007). Deletion of *HSP31* led to increased levels of ROS and to hypersensitivity against oxidative stress independent of the respiratory competence of the yeast strains (clonogenic survival assays) (Skoneczna et al., 2007). The cellular levels of Hsp31 were increased upon stress, dependent on the oxidative stress response transcription factor Yap1 (Skoneczna et al., 2007). These data suggest that Hsp31 is an oxidative stress-induced chaperone that ameliorates cytotoxicity elicited by elevated levels of ROS. Although Hsp31 expression did not relieve Lrrk2-triggered cytotoxicity in yeast (growth assays) (Xiong et al., 2010), it is of interest whether Hsp31 is able to modulate cytotoxicity elicited by α-synuclein expression.

#### Yeast PD models may be used to elucidate the role of diverse mitochondrial quality control mechanisms in modulating cytotoxicity and cell death

Mitochondrial dysfunction is a common mechanism underlying the pathogenesis of both sporadic and familiar forms of PD (Correia et al., 2012). In PD patients and animal PD models, activities of complex I of the mitochondrial respiratory chain were significantly decreased (Correia et al., 2012). The importance of complex I deficiency can be estimated by the fact, that pharmaceutical treatment of animal models and humans with complex I inhibitors, including MPTP and rotenone, was sufficient for the loss of dopaminergic neurons, and consequently elicited clinical symptoms typical for PD (Correia et al., 2012). *S. cerevisiae* cannot be used as model for analyzing complex I dysfunction, because it lacks a complex I ortholog (Li et al., 2006). Instead three enzymes, one internal NADH dehydrogenase (Ndi1), and two external NADH dehydrogenases (Nde1 and Nde2), accomplish the function of mammalian complex I (Li et al., 2006). Notably, yeast Ndi1 can fully complement complex I deficiency in mammalian cells, and therefore Ndi1 expression has been suggested to cure complex I dysfunction in PD patients (Marella et al., 2009).

Consistent to the mentioned complex I deficiency, mtDNA encoded defects, increased levels of ROS, and a decrease in the mitochondrial membrane potential could be observed in PD patients, animal and cell culture PD models (Correia et al., 2012). The observed increased susceptibility for mitochondrial permeabilization points to mitochondrion-specific cell death (Correia et al., 2012). In yeast cells expressing α-synuclein, increased levels of mitochondrially produced ROS (Flower et al., 2005; Büttner et al., 2008; Su et al., 2010), decreased mitochondrial membrane potential (Lee et al., 2008), and release of Cyt. *c* into the cytosol were observed (Flower et al., 2005). These data suggest that aberrant mitochondrial respiratory chain activities lead to ROS production and mitochondrial permeabilization in yeast, i.e., similar events that occur in PD patients.

Increased mitochondrial degradation via mitophagy was observed in PD patients and animal PD models (De Castro et al., 2011). Notably, the PD genes encoding PINK1 and parkin promote mitophagy of damaged mitochondria (Geisler et al., 2010; Springer and Kahle, 2011; Jin and Youle, 2012). PINK1 is a mitochondrial kinase, which serves as a sensor of damaged and depolarized mitochondria. In healthy mitochondria, PINK1 is imported into the organelles, whereas in depolarized mitochondria it rapidly accumulates at the mitochondrial outer membrane with its kinase domain facing to the cytosol. Here, it recruits the E3 ligase parkin, which ubiquitylates the mitochondrial fusion proteins mitofusin 1 and 2, initiating mitochondrial fragmentation and mitophagy. Since PD-associated mutations of PINK1 and parkin interrupted this pathway, these data suggest that in PD the accumulation of damaged mitochondria due to an impairment of mitophagy contributes to PD pathogenesis (Geisler et al., 2010). This model is underlined by the fact that PD-associated mutations in the PD gene encoding ATP13A2 also resulted in lysosomal dysfunction, which also impairs autophagic pathways (Dehay et al., 2012).

Whereas in mammalian PD models, mitophagy appears to play a beneficial role, the data observed in yeast PD models suggest for a lethal function of mitophagy: mitophagy is lethal during cell death upon expression of α-synuclein in yeast (Sampaio-Marques et al., 2012), and the expression of Lrrk2 fragments in yeast triggered a marked increase in autophagic vacuoles hinting to a disturbance of autophagic processes (Xiong et al., 2010). Future studies will be needed to address the obvious discrepancy between the data obtained in mammalian and yeast PD models. It is tempting to speculate that, in the first instance, mitophagy plays as *per se* cytoprotective role by removing damaged mitochondria, which can be very harmful for cell survival. However, it is thinkable that upon overload of this pathway, the process of mitophagy may get inefficient and cytotoxic. In an alternative scenario, the efficiency of mitophagy may be too high, and as consequence too many healthy mitochondria are removed from the cell. Yeast cells could tolerate a severe loss of healthy mitochondria, because they can easily switch from respiration to fermentation. However, since neurons essentially depend on high rates of respiration, they would severely suffer from the loss of healthy mitochondria.

### Huntington disease (HD)

HD is an autosomal dominant neurodegenerative disorder characterized by a progressive loss of neurons in the striatum and the cortex with a consequent decline of cognitive and motor functions (Ross and Tabrizi, 2011). HD is caused by an abnormal polyglutamine (polyQ) expansion in the protein huntingtin due to an aberrant CAG codon expansion in the exon 1 of the gene encoding huntingtin (Ross and Tabrizi, 2011). This results in an aggregation-prone protein eventually triggering cytotoxicity and neuronal cell loss (Ross and Tabrizi, 2011). Increasing the length of the polyQ expansion accelerates aggregation of huntingtin and strictly correlates with the increase in cytotoxicity and the decrease in disease onset (Ross and Tabrizi, 2011). In order to dissect underlying mechanisms, various disease HD models have been established, comprising transgenic HD mouse, mammalian cell culture, and yeast (Bossy-Wetzel et al., 2008; Mason and Giorgini, 2011; Ross and Tabrizi, 2011; Correia et al., 2012).

#### Expression of disease-associated huntingtin in yeast results in cytotoxicity, cell death, and critical mitochondrial damage

Yeast HD models expressing GFP-fusion constructs of exon 1 of human huntingtin comprising various lengths of polyQ expansion were generated (Meriin et al., 2002; Duennwald et al., 2006; Solans et al., 2006; Ocampo et al., 2010). PolyQ constructs encoding 103 glutamine residues (103Q) efficiently triggered growth deficits and apoptotic cell death (Sokolov et al., 2006; Solans et al., 2006; Ocampo et al., 2010) (Table 1). In contrast, polyQ constructs encoding 25 glutamine residues (25Q) remained nontoxic, and the constructs with intermediate polyQ lengths showed intermediate cytotoxicity (Sokolov et al., 2006; Solans et al., 2006; Ocampo et al., 2010). Upon 103Q expression, the mitochondrial network disrupted, the mitochondrial membrane potential dissipated, the mitochondrial protein synthesis efficiency decreased, correlated with markedly reduced respiratory rates and defective energetic coupling (Solans et al., 2006; Ocampo et al., 2010). The defects in respiratory capacity were a result of impaired activities and decreased steady-state levels of the respiratory chain complexes II and III (Solans et al., 2006). As consequence of the impairment of the respiratory chain increased ROS levels were observed (Sokolov et al., 2006; Solans et al., 2006). Treatment with the antioxidants α-tocopherol and resveratrol relieved the level of mitochondrial fragmentation and reduced respiratory impairment, but failed to decrease cytotoxicity (growth assays) or increase cell survival (Sokolov et al., 2006; Solans et al., 2006). These data suggest that ROS are not essential for cell death but may accelerate cytotoxicity in a vicious cycle by damaging the iron-sulfur clusters of the respiratory chain, which are very susceptible for oxidative damage, culminating in the production of increased ROS levels (Solans et al., 2006). Mitochondria isolated from yeast cultures expressing polyQ (103Q) demonstrated altered osmotic properties and facilitated mitochondrial permeabilization (Ocampo et al., 2010), which is characteristic for mitochondrion-dependent cell death in yeast and mammalian cells (Galluzzi et al., 2009; Guaragnella et al., 2012). Thus, critical mitochondrial damage upon polyQ expression in yeast induces mitochondrion-dependent cell death.

#### Critical mitochondrial damage in yeast HD models is due to direct and indirect mechanisms

Some polyQ aggregates directly interacted with mitochondria (Solans et al., 2006) and polyQ domains localized to the mitochondrial outer membrane (Ocampo et al., 2010). PolyQ aggregates sequestered the mitochondrial proteins Ilv5, which is involved in mtDNA maintenance, Atp2, which is a component of complex V of the mitochondrial respiratory chain, and Ptk1, a mitochondrial protein kinase (Wang et al., 2009). These data suggest that at least part of polyQ-triggered cytotoxicity is due to a direct damage of mitochondria (Mason and Giorgini, 2011). In addition, polyQ-triggered cytotoxicity could impair mitochondria in more indirect pathways. For instance, polyQ expression resulted in destabilization of the actin cytoskeleton (Solans et al., 2006), which is in yeast essential for mitochondrial motility and the maintenance of the mitochondrial network, and therefore is pivotally linked with mitochondrial function (Frederick and Shaw, 2007). PolyQ expression also drastically impaired the ubiquitin-dependent ER-associated protein degradation pathway (ERAD) by sequestering the pivotal ERAD components Cdc48 and Npl4 (Duennwald and Lindquist, 2008). This resulted in the induction of the unfolded protein response and ER stress (Duennwald and Lindquist, 2008). Since ER and mitochondria are physically and functionally linked, *e.g*., the ER is pivotally involved in the turnover of phospholipids to and from the mitochondrial membranes (Fujimoto and Hayashi, 2011), ERAD dysfunction due to polyQ expression may indirectly result in the damage of mitochondrial membranes (Braun and Zischka, 2008). Recently, a new ubiquitin-dependent protein quality control mechanism on the mitochondrial outer membrane was identified (Heo et al., 2010; Taylor and Rutter, 2011). This so-called mitochondrion-associated protein degradation pathway (MAD) shares the components Cdc48 and Npl4 with the ERAD pathway (Heo et al., 2010; Taylor and Rutter, 2011). Therefore, it is tempting to speculate that polyQ expression also impairs MAD, culminating in a more direct damage of the mitochondrial outer membrane.

#### Stimulation of mitochondrial biogenesis and repair pathways can cure critical mitochondrial damage and cytotoxicity in yeast HD models

Overexpression of *HAP4*, encoding the catalytic subunit of the transcriptional activator complex triggering mitochondrial biogenesis, reduced growth deficits upon polyQ expression, ameliorated the cellular respiratory defects, restored mitochondrial membrane potential, and increased mitochondrial protein translation efficiency (Ocampo et al., 2010). Expression of polyQ in three different knock-out strains, which relieved polyQ-triggered cytotoxicity (growth assays), resulted in the induction of the gene encoding D-lactate dehydrogenase (Dld3) (Tauber et al., 2011). In contrast, expression of polyQ in a yeast strain deleted for the peroxisomal citrate synthase (Cit2) dramatically increased polyQ-triggered cytotoxicity (growth assays) (Willingham et al., 2003). Both Dld3 and Cit2 are induced upon mitochondrial damage and are part of the retrograde response, which is a cellular defense mechanism to counteract mitochondrial damage (Liu and Butow, 2006). Therefore, the mitochondrial retrograde response and mitochondrial biogenesis could play protective roles in relieving mitochondrial damage and polyQ-triggered cytotoxicity.

#### Mitochondrion-associated metabolites contribute to cytotoxicity in yeast HD models

A genome-wide loss-of-function suppressor screen, in which gene deletions were identified, that suppress cytotoxicity of polyQ (growth assays), resulted in the identification of another mitochondrion-associated pathway, modulating polyQ-triggered cytotoxicity (Giorgini et al., 2005). Deletion of the *BNA4* gene, encoding the mitochondrial enzyme kynurenine 3-monooxygenase, involved in the kynurenine pathway of tryptophan degradation and NAD^+^ synthesis, resulted in the most potent suppression of polyQ-triggered cytotoxicity (growth assays) (Giorgini et al., 2005). Whereas *bna4*Δ cells lacked the intermediates 3-hydroxykynurenine and quinolinic acid of the kynurenine pathway, wild-type cells expressing polyQ accumulated these metabolites and induced expression of enzymes of the kynurenine pathway (Giorgini et al., 2005, 2008; Tauber et al., 2011). Treatment of polyQ-expressing yeast cells with an inhibitor of Bna4 reduced the levels of the metabolites and reduced polyQ-triggered cytotoxicity (growth assays) and ROS production (Giorgini et al., 2005). Consistently, 15 of the 28 identified yeast knock-out strains, which suppressed polyQ-triggered cytotoxicity (growth assays), demonstrated significantly decreased levels in 3-hydroxykynurenine and quinolinic acid (Giorgini et al., 2008). These data indicate that accumulation of these metabolites contributes to polyQ-triggered cytotoxicity (Mason and Giorgini, 2011). However, how accumulation of these metabolites triggers mitochondrial damage remains unknown.

#### Yeast HD models recapitulate mitochondrial respiratory and metabolic defects

Expression of disease-associated huntingtin is associated with critical mitochondrial dysfunction in HD patients, in transgenic murine and mammalian cell culture HD models (Bossy-Wetzel et al., 2008; Ross and Tabrizi, 2011; Correia et al., 2012). Bioenergetic defects, impaired activity of the respiratory chain complexes II and III, depolarization of mitochondrial membrane potential, fragmentation of the mitochondrial network, high levels of oxidative stress, and accumulation of the intermediates 3-hydroxykynurenine and quinolinic acid of the kynurenine pathway are characteristic for the progression of HD (Reynolds and Pearson, 1989; Bossy-Wetzel et al., 2008; Ross and Tabrizi, 2011; Correia et al., 2012). Since these hallmarks could also be identified in yeast HD models, yeast appears to be an appropriate model organism to elucidate pathological mechanisms on mitochondria upon polyQ-triggered cytotoxicity. Further key findings in yeast are that the stimulation of mitochondrial biogenesis and repair pathways and genetic and pharmacological manipulation of the kynurenine pathway can markedly relieve cytotoxicity upon polyQ expression. Indeed, transcriptional regulation of the kynurenine pathway and its role in modulating cytotoxicity upon polyQ expression is very similar in yeast and in transgenic HD mouse models (Giorgini et al., 2008; Campesan et al., 2011; Zwilling et al., 2011). Therefore, yeast may facilitate the discovery of pathological pathways, which can be exploited for new therapeutic approaches for HD treatment.

In yeast HD models, mitochondrion-dependent molecular cell death could be discriminated between Cyt. *c*-, apoptosis-inducing factor-, and endonuclease G-dependent or alternative scenarios. Further, yeast HD models could be applied for elucidating the role of mitochondrial fusion, fission, morphology and motility, and for describing the maintenance of mtDNA. Mitochondrial permabilization concomitant with mitochondrion-dependent cell death and damage of mtDNA are hallmarks of HD (Bossy-Wetzel et al., 2008; Correia et al., 2012). Inhibiting mitochondrial fission and promoting mitochondrial fusion has recently been described to relieve polyQ-triggered cytotoxicity in mammalian cells (Song et al., 2011). Therefore, a more detailed analysis in yeast HD models could be helpful for better understanding the roles of these conserved pathways in HD.

Autophagy is involved in the degradation of huntingtin aggregates (Qin et al., 2003), and mitophagy has been described to occur in immortalized cell lines from HD patients (Mormone et al., 2006). Different huntingtin domains demonstrate homology to the yeast autophagy proteins Atg23, Vac8, and Atg11, raising the question whether huntingtin itself is involved in autophagy/mitophagy regulation (Steffan, 2010). Therefore, analyzing the role of mitophagy in modulating polyQ-triggered cytotoxicity in yeast is also of high interest.

### Amyotrophic lateral sclerosis (ALS)

ALS is a frequent degenerative motor neuron disease characterized by adult-onset loss of the lower and upper motor neuron systems, resulting in muscle weakness and wasting (Andersen and Al-Chalabi, 2011). Mutations in 13 different genes are causative for hereditary forms of ALS (Andersen and Al-Chalabi, 2011; Wu et al., 2012), and even more genes are putatively associated with ALS (Andersen and Al-Chalabi, 2011). The most common ALS-associated genes are *SOD1*, *TARDBP*, and *FUS*, encoding the ROS-scavenger enzyme superoxide dismutase 1 (SOD1), and the RNA-binding proteins TDP-43 and FUS/TLS (Andersen and Al-Chalabi, 2011). ALS-associated variants of these proteins demonstrate mislocalization and/or a high tendency for aggregation (Andersen and Al-Chalabi, 2011; Da Cruz and Cleveland, 2011). Yeast models expressing ALS-associated wild-type and mutant SOD1, TDP-43, and FUS/TLS have been established to further analyze the detrimental roles of these proteins on cell survival (Bastow et al., 2011).

#### ALS-associated mutant Sod1 variants demonstrate increased mitochondrial localization and impair mitochondrial respiratory chain activity

Yeast cells lacking *SOD1*, the ortholog of human *SOD1*, are sensitive against oxidative stress (growth assays), highlighting the role of Sod1 as superoxide scavenger (Bastow et al., 2011). Sod1 is also involved in the assembly of iron-sulfur clusters, which are important for enzymes of the respiratory chain, the TCA cycle, and amino acid biosynthetic pathways (Strain et al., 1998; Wallace et al., 2004; Sehati et al., 2011). Sod1 demonstrates dual localization between the cytosol, where the vast majority of Sod1 is localized, and the mitochondrial intermembrane space (Klöppel et al., 2010). Surprisingly, expression of ALS-associated mutant (G93A) Sod1 in yeast resulted in an increased mitochondrial localization and an increased tolerance against mitochondrially produced ROS (Klöppel et al., 2010) (Table 1). Notably, a decrease in electron transport in the mitochondrial respiratory chain was observed upon expression of ALS-associated mutant (G93A) Sod1 (Gunther et al., 2004). Therefore, it is tempting to speculate that increased mitochondrial localization of Sod1 disturbs the mitochondrial respiratory chain, and that this mechanism contributes to cell loss (Bastow et al., 2011). Both activation and mitochondrial localization of Sod1 depend on the copper chaperone Ccs1, which demonstrates high sequence identity with human SOD1 (Leitch et al., 2009; Groß et al., 2011; Klöppel et al., 2011). ALS-associated mutant Sod1 variants were rapidly degraded in yeast cells lacking *CCS1* (Carroll et al., 2006), underlining the assumption that their mitochondrial localization is important for their detrimental action.

#### Expression of TDP-43 and FUS/TLS are cytotoxic in yeast, and mitochondrial respiratory capacity plays a pivotal role in modulating TDP-43-triggered cytotoxicity

Expression of wild-type and disease-associated mutant variants of TDP-43 and FUS/TLS triggered cytoplasmic protein aggregation leading to growth deficits, loss of clonogenic survival, and apoptosis and necrosis in yeast (Johnson et al., 2008, 2009; Elden et al., 2010; Armakola et al., 2011; Braun et al., 2011; Couthouis et al., 2011; Fushimi et al., 2011; Ju et al., 2011; Kryndushkin et al., 2011; Robinson, 2011; Sun et al., 2011) (Table 1). TDP-43-expressing yeast cells showed an age-dependent loss of clonogenic survival concomitant to ROS accumulation (Johnson et al., 2008; Braun et al., 2011). Mitochondria play a pivotal role in executing TDP-43-triggered cell death (clonogenic survival assays): (1) Cytotoxicity was significantly decreased in cells lacking mtDNA and functional mitochondria, (2) impairment of the respiratory chain relieved cytotoxicity with a stringent correlation between cytotoxicity and the degree of respiratory capacity or mtDNA stability, and (3) increasing the respiratory capacity enhanced TDP-43-triggered ROS production and cell death (Braun et al., 2011). Mitochondrion-dependent cell death was independent of the mitochondrial cell death proteins apoptosis-inducing factor, endonuclease G, and Cyt. *c*, suggesting that an alternative mitochondrion-dependent cell death pathway may be active (Braun et al., 2011).

Whether FUS/TLS-triggered cytotoxicity is also modulated by mitochondrial function is still an open question. However, in a genome-wide screen to identify modulators of FUS/TLS-triggered cytotoxicity (growth assays) upon overexpression or deletion, several mitochondrion-associated candidate genes were identified (Sun et al., 2011). Yeast strains deleted for genes encoding components of the mitochondrial protein translation machinery, the mitochondrial respiratory chain, and the TCA cycle increased FUS/TLS-triggered cytotoxicity (growth assays) (Sun et al., 2011). These data suggest that impaired mitochondria upon deletion of these genes are more vulnerable against FUS/TLS expression, and raise the possibility that mitochondrial damage contributes to the execution of FUS/TLS-triggered cytotoxicity.

#### Yeast ALS models may support and complement hypotheses obtained in higher model systems

Structural mitochondrial abnormalities as seen in electron microscopy and markers of oxidative stress were observed in ALS patients, and in mouse models expressing ALS-associated mutant variants of SOD1 and TDP-43 (Martin, 2011; Cozzolino et al., 2012). In ALS patients with SOD1 etiology Cyt. *c* release correlated with apoptosis, and in mouse ALS models for SOD1-associated ALS, mitochondrial permeabilization correlated with necrosis (Martin, 2011). These data hint to the induction of mitochondrion-specific cell death during ALS. In yeast, expression of TDP-43 triggered oxidative stress, and apoptosis and necrosis, and mitochondrial respiration was found to be an important modulator of cytotoxicity (clonogenic survival assays) (Braun et al., 2011). Thus, the determination and critical comparison of cell death pathways in yeast cells expressing ALS-associated wild-type and mutant SOD1, TDP-43, and FUS/TLS may help to elucidate common and distinct mechanisms underlying cytotoxicity and mitochondrion-dependent cell death with potential relevance for ALS.

Abnormally aggregated mitochondria, as seen in mouse models for TDP-43 and FUS/TLS-mediated ALS, suggest for deficits in mitochondrial trafficking in motor neurons, a phenomenon which has already been proven in mouse models expressing ALS-associated mutant SOD1 (Martin, 2011; Cozzolino et al., 2012). Expression of ALS-associated mutant SOD1, TDP-43, and FUS/TLS triggered mitochondrial fragmentation in mouse motor neurons, suggesting a further role of the mitochondrial fusion/fission balance in modulating pathogenesis (Magrane et al., 2009, 2012; Xu et al., 2010, 2011; Cozzolino et al., 2012; Tradewell et al., 2012). The mechanisms of mitochondrial fusion/fission are conserved from humans to yeast (Westermann, 2010). Therefore, yeast ALS models may help to dissect components of the mitochondrial fusion/fission machinery, which pivotally contribute to the modulation of cytotoxicity. In contrast, for analyzing the role of mitochondrial trafficking in ALS pathology, yeast ALS models might be of limited use, because the mitochondrial trafficking in yeast depends on the actin cytoskeleton, whereas neuronal mitochondria are transported along the microtubule cytoskeleton (Frederick and Shaw, 2007).

ALS-associated mutant SOD1 demonstrated increased mitochondrial association, which is correlated with elevated ROS production and pathology (Martin, 2011; Cozzolino et al., 2012). In contrast, mitochondrial aggregates in mouse models expressing TDP-43 and FUS/TLS did not contain the disease-causing proteins, suggesting that the detrimental effects of these proteins for mitochondria are rather indirect (Xu et al., 2010; Cozzolino et al., 2012). Consistent to the observations in mouse models, ALS-associated mutant (G93A) SOD1 demonstrated increased localization in the mitochondrial intermembrane space in yeast (Klöppel et al., 2010), with potential harmful effects for mitochondrial respiratory chain efficiency (Gunther et al., 2004). Due to the extensive knowledge on the regulation of mitochondrial import of SOD1 in yeast (Leitch et al., 2009; Groß et al., 2011; Klöppel et al., 2011), yeast might be a good model to elucidate molecular components and pathways involved in SOD1 pathology. In yeast, TDP-43 aggregates demonstrated a peri-mitochondrial localization, although a strong interaction between TDP-43 and mitochondria could not be observed (Braun et al., 2011). These data suggest that in yeast, as in humans, indirect effects may contribute to the pivotal mitochondrial damage. The elucidation of these mechanisms of mitochondrial damage will be one interesting future task.

### Other neurodegenerative disorders

Polyalanine (polyA) disorders are associated with trinucleotide repeat expansions in genes involved in developmental processes, including central nervous system development (*ZIC2*), neural development (*SOX3*), and cerebral development and patterning (*ARX*) (Messaed and Rouleau, 2009). The trinucleotide repeat expansions encode abnormal polyA expansions causing protein aggregation and neuronal loss, and therewith triggering neurodegenerative diseases, such as holoprosencephaly, X-linked hypopituitarism, and X-linked mental retardation (Messaed and Rouleau, 2009). Recently, a yeast model for polyA-triggered cytotoxicity has been established (Konopka et al., 2011). With increasing length of the polyA stretches, cytotoxicity markedly increased, as measured by growth deficits and decreased plasma membrane integrities (Konopka et al., 2011). Whether mitochondria are affected and pivotally involved in polyA-triggered cytotoxicity in yeast remains unknown. However, mitochondrial dysfunction, the formation of the mitochondrial permeability transition pore, Cyt. *c* release, and the translocation of pro-apoptotic BAX to mitochondria have been observed in mammalian polyA models (Toriumi et al., 2008, 2009; Bhattacharjee et al., 2012). Therefore, it might be a fruitful approach to dissect the role of mitochondria in polyA-triggered cytotoxicity in yeast.

Prion disorders are fatal neurodegenerative conditions characterized by the conversion of the soluble cellular protein PrP^C^ into its insoluble and infectious prion form PrP^Sc^ (Imran and Mahmood, 2011). For instance in Creutzfeld-Jakob disease, PrP^Sc^ accumulation results in neuronal loss causing rapid progressive dementia and cerebellar dysfunction (Imran and Mahmood, 2011). Yeast models have been established expressing mammalian PrP^C^ (Li and Harris, 2005; Bounhar et al., 2006; Halfmann et al., 2010; Josse et al., 2012). PrP^C^ expression was not cytotoxic for yeast cells (Li and Harris, 2005; Bounhar et al., 2006). In contrast, PrP^C^ expression suppressed yeast cytotoxicity triggered by the expression of the mammalian pro-apoptotic BAX, as measured by growth assays, clonogenic survival assays, and assays measuring metabolic activities (Li and Harris, 2005; Bounhar et al., 2006). Consistently, PrP^C^ expression prevented BAX-induced cell death in primary neuronal cultures (Bounhar et al., 2001). Since BAX triggers mitochondrion-dependent cell death in mammalian cells and in yeast (Khoury and Greenwood, 2008), these data suggest that PrP^C^ plays a protective role in preventing mitochondria from executing cell death (Li and Harris, 2005; Bounhar et al., 2006).

## Conclusions and outlook

Mitochondrial damage and dysfunction are hallmarks of AD, PD, HD, and ALS (Martin, 2011; Correia et al., 2012; Cozzolino et al., 2012). Yeast models for the elucidation of mechanisms of cytotoxicity and cell death upon expression of AD-, PD-, HD-, and ALS-associated proteins have been established (Gitler, 2008; Miller-Fleming et al., 2008; Winderickx et al., 2008; Bharadwaj et al., 2010; Braun et al., 2010; Khurana and Lindquist, 2010; Bastow et al., 2011; De Vos et al., 2011; Mason and Giorgini, 2011). In these models mitochondrial (dys)function contributes to cytotoxicity. The description of mitochondrial damage, the elucidation of mitochondrion-specific cell death pathways, and the dissection of the roles of mitochondrial quality control mechanisms, including mitochondrial dynamics and degradation, has just initiated in these yeast models. Therefore, neurotoxic yeast models will elucidate novel paradigms of mitochondrial pathobiology, which can be easily validated in other model organisms for neurodegenerative disorders.

### Conflict of interest statement

The author declares that the research was conducted in the absence of any commercial or financial relationships that could be construed as a potential conflict of interest.
